# Factors Controlling Vegetation Fires in Protected and Non-Protected Areas of Myanmar

**DOI:** 10.1371/journal.pone.0124346

**Published:** 2015-04-24

**Authors:** Sumalika Biswas, Krishna Prasad Vadrevu, Zin Mar Lwin, Kristofer Lasko, Christopher O. Justice

**Affiliations:** 1 Department of Geographical Sciences, University of Maryland, College Park, Maryland, United States of America; 2 Mandalay Technological University, Patheingyi, Mandalay, Myanmar; University of Calgary, CANADA

## Abstract

Fire is an important disturbance agent in Myanmar impacting several ecosystems. In this study, we quantify the factors impacting vegetation fires in protected and non-protected areas of Myanmar. Satellite datasets in conjunction with biophysical and anthropogenic factors were used in a spatial framework to map the causative factors of fires. Specifically, we used the frequency ratio method to assess the contribution of each causative factor to overall fire susceptibility at a 1km scale. Results suggested the mean fire density in non-protected areas was two times higher than the protected areas. Fire-land cover partition analysis suggested dominant fire occurrences in the savannas (protected areas) and woody savannas (non-protected areas). The five major fire causative factors in protected areas in descending order include population density, land cover, tree cover percent, travel time from nearest city and temperature. In contrast, the causative factors in non-protected areas were population density, tree cover percent, travel time from nearest city, temperature and elevation. The fire susceptibility analysis showed distinct spatial patterns with central Myanmar as a hot spot of vegetation fires. Results from propensity score matching suggested that forests within protected areas have 11% less fires than non-protected areas. Overall, our results identify important causative factors of fire useful to address broad scale fire risk concerns at a landscape scale in Myanmar.

## Introduction

Fire is a common land management tool in the tropics [1], [2], [3]. Traditionally, fires have been used for slash and burn agriculture in the denser forests and to clear the forest floor in the open forests [3]. However, in recent times, population growth and economic incentives for agriculture have led to widespread clearing of tropical forests to meet food security and urban housing demands [4], [5]. Clearing of forests for agriculture has led to fragmentation of the forests with increased forest edges resulting in greater fire risk [6], [7]. In addition, changing climatic patterns and human land use also alters fire regimes in the tropics [8], [9], [10].

Specific to Asia, the mosaicked forested landscape of mainland SE Asia harbors a wide range of habitats and is a part of the Indo-Burma biodiversity hotspot [11], [12], [13]. The region mostly comprises fire sensitive forests like tropical broadleaf deciduous forests. The patches of moist broadleaf forests are often interspersed with fire-dependent dry seasonal broadleaf forests [14]. Literature review suggests that open canopy forests are more prone to fire than closed forests as the floor in open forests is drier than closed forests due to greater degree of sunlight penetration [15]. Fire susceptibility is also dependent on land cover type. Shrublands are known to be more flammable than evergreen forests [16]. Generally, as the agricultural frontier expands at the expense of forest lands, the forest patches near the edges are cleared before the forest interior patches. Also, agricultural fires can spread to nearby forests accidentally. Thus, in cases involving anthropogenic fires, the distance to forest edge is expected to be inversely proportional to fire occurrence [1], [6], [15].

Apart from vegetation, the topographic factors also play a role in fire susceptibility. Elevation, slope and aspect influence fire behavior [17], [18]. Elevation influences fire spread by impacting the wind behavior and precipitation patterns [19]. Low elevation areas are more prone to fire due to higher temperature, less precipitation and settlements [20], [21]. Fire spreads upslope faster as heat rises and preheats the upslope fuel [22]. The aspect is the compass direction that a slope faces. It determines the amount of insolation, and precipitation received by the surface; the directions which receive more heat and less moisture are more prone to fire. Among climatic factors, temperature is a major factor because it is known that warmer regions are more susceptible to fires than colder regions. Higher air temperatures increases the surface temperature and dry the fuel, increasing the probability of fire [9], [10], [23].

Literature review suggests most tropical fires as anthropogenic in nature [4],[6], [24], [25]. Distance from road is a measure of accessibility to forest. Forest patches near the road are more prone to disturbance than forest patches deep inside forests which are not easily accessible [26], [27]. Roads provide a transportation route for agricultural goods and timber. Since fires are used to clear forests in the tropics, we assumed that distance to roads would be an important anthropogenic causative factor [27], [28]. Accessibility from cities is a proxy for distance to markets. Earlier studies have shown that the probability of forest clearing near cities is higher due to easily accessible markets to sell the produce or easy transportation facilities [29], [30].

Among the Asian nations, Myanmar is reported to have the highest number of vegetation fires [31]; however, the causative factors largely remain uninvestigated. In the past, political isolation and poor data availability has made Myanmar a very difficult region for studying environmental problems. In recent times, the country’s changed political system has opened the doors for environmental research. In this study, we focused on causative factors of fires in protected and non-protected areas of Myanmar.

**Fig 1:**
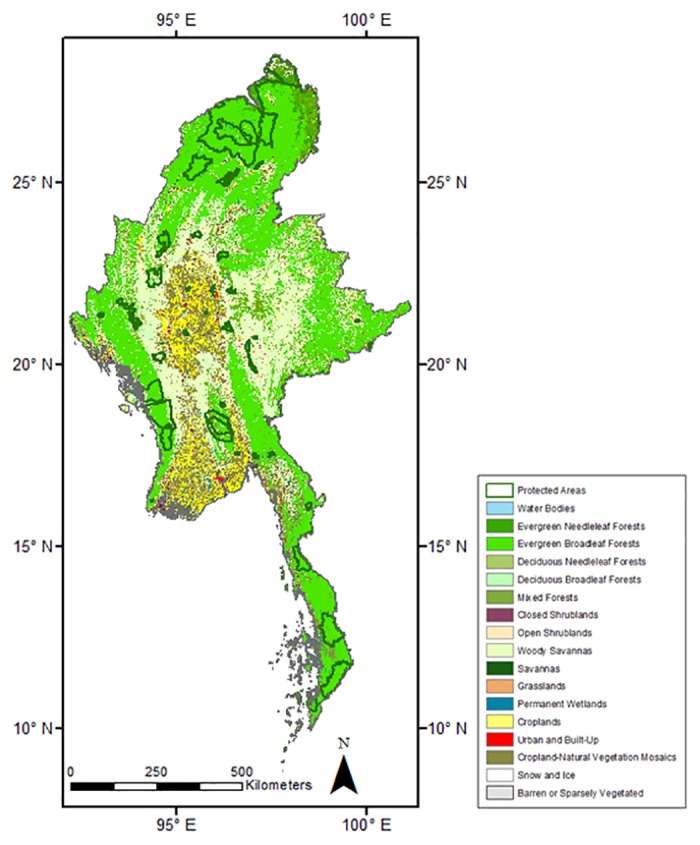
A map showing the protected areas in Myanmar with boundaries in dark green color. MODIS land cover map is shown in the background.

In Myanmar, for the past 20 years, conservation efforts have largely focused on increasing protection in the forested areas [3]. Previously, researchers have documented the role of protected areas, location and strength of management influencing conservation efforts in different countries [32], [33], [34], [35], [36], [37], [38]. However, no such attempt has been made for Myanmar. Very little is known about the effectiveness of the protected areas in Myanmar. Extending from north to south in Myanmar, the protected areas conserve a wide range of tropical forests harboring rich biodiversity (Fig 1). Due to Myanmar’s history of economic isolation, large tracts of its forests and associated biodiversity seems to be conserved, as compared to its neighboring countries [39]. Traditionally, factors posing threats to the forests of Myanmar include hunting and slash and burn agriculture [40]. However, clearing of forests for large scale industrial agriculture, infrastructure developments, uncontrolled exploitation of natural resources seem to pose a bigger risk in recent times [41], [42]. A review of country reports suggests that protecting the forests has always been a difficult task given the limited financial and technical resources available to the protected area managers in Myanmar. Nevertheless, the status of conservation of most protected areas seems good though some regions are doing better than others [43].

Rapid developments in computing and information technology including remote sensing have made a wide variety of data sets available to study environmental problems including fires [44]. Specific to fires, MODIS Active Fire data provide the geolocation of fires within 24 hours of fire occurrence at a global scale [45], [46]. The characteristic spectral and temporal resolution of MODIS makes it ideal for fire detection [47], [48]. In addition to fires, recent advances in forest mapping have also enabled the production of high resolution, spatially consistent forest cover maps at a global scale [49]. The high spatial resolution has made it possible to capture small-scale forest losses which were previously not possible. The high resolution fire and forest extent data can provide a unique opportunity to study vegetation fire characteristics. Further, geospatial technologies can be effectively used to compute ancillary variables of anthropogenic factors impacting fires such as distance to roads, forests, urban dwellings, etc. In this study, we integrate both biophysical data (satellite data on fires, land cover, elevation, slope, tree percent, etc.) as well as anthropogenic factors (population density, distance travelled to nearest forests, distance to roads) to quantify causative factors of fires in protected and non-protected areas.

In the tropics, the role of protected areas as a tool of conservation is highly debatable [32], [33], [35], [38], [50], [51]. Yet, most researchers agree that at the broader scale, protected forests are better conserved than the unprotected forests though different levels of governance may influence the degree of protection [32], [33], [34], [35]. The question of effectiveness is very relevant to Myanmar because of the 43 protected parks of which 17 are reportedly paper parks [43]. As of 2009, 10.1–15.5% of the global land surface is conserved in protected areas [52]. Since fire is a disturbance agent in the tropics, the probability of fires inside protected areas is expected to be less compared to non-protected areas mainly due to fire suppression strategies and legal mechanisms [32].

In this study, we address several questions relevant to vegetation fires in protected and non-protected areas of Myanmar. a) What are the dominant causative factors of fires in protected and non-protected areas? b) Are the causative factors the same or different for different regions? c) How do the causative factors of fires vary spatially in protected and non-protected areas and where are the hotspots? d) What is the relative role of each causative factor in controlling fires? We address the above questions using a probabilistic frequency analysis approach integrating different biophysical and anthropogenic datasets. We hypothesized that protected areas will have fewer fires than non-protected areas. Accordingly, we also tested the relative importance of protected areas compared to non-protected areas in fire prevention and conservation using the propensity score matching technique.

## Material and Method

### Study Area

Myanmar (formerly known as Burma) is located in mainland SE Asia, between 9°32’N to 28°31’N and 92°10’E and 101°11’E. It covers an area of 676,580 km^2^. The landscape is highly heterogeneous in terms of topography. The mountainous regions are found in northern, western parts of the country while the Shan plateau is located in the east. The central region of the country consists of plains and is the seat of agriculture. The climate is heavily influenced by the south west Asian Monsoon. Myanmar has two marked seasons, dry season (November to April) and wet season (May to October). The mean annual rainfall varies considerably and is dependent on the local topography. Monthly precipitation of dry season (Nov. to Apr.) is less than 100mm. Based on the temperature differences, the dry season can be divided into the hot-dry (March-April) and cold-dry (November to February) period. The monthly mean temperature of April, the hottest month exceeds 33°C. March and April correspond to the severe fire months. Forests cover 48% of mainland Myanmar [53]. The major forest type is mixed deciduous (38%) followed by tropical evergreen forests (16%) [54]. Broadleaf evergreen forests dominate in northern and southern Myanmar while broadleaf deciduous forests are found mostly in the central, mountainous region. A land cover classification map of Myanmar is shown in Fig 1. Fire is an important disturbance agent in the forests of Myanmar. Every year a large area of forests is lost to fires resulting in habitat loss and degradation of forests. The highest number of fires occur in the month of March. In the past decade (2000–2012), 2007 year was found to have highest number of fires. Though the forests of Myanmar is plagued by fires, the causative factors are not clearly documented. Besides the natural factors like climate and fuel characteristics together with increased anthropogenic pressure are expected to contribute to the high numbers of fires in the country. Establishing protected areas have been proposed to be a successful way to conserve forests. Most of the forests are outside protected areas, though efforts are being made by the new government to increase the number of protected areas and extend the boundaries of a few existing protected areas [43]. Myanmar has 43 officially recognized protected areas covering 7.3% of the country. The location of protected areas in Myanmar is shown in Fig 1. Of the 43 protected areas, 17 are reportedly paper parks. 76 Key Biodiversity Areas (KBAs) have been identified so far, including 54 Important Bird Areas (IBAs) and the Natma Taung National Park as an area of local endemism [43].

### Data

We used a variety of datasets for quantifying the major causative factors of fires. The active fire locations were obtained from the MODIS/Aqua & Terra Thermal Anomalies/Fire locations 1km Collection 5.1 dataset [46], also known as MCD14ML. The product is created by the University of Maryland as a monthly fire product and distributed through NASA LANCE-FIRMS website (https://earthdata.nasa.gov/data/near-real-time-data/firms) [45]. The dataset provides geolocation, brightness, scan and track, date, time, sensor, confidence and version for each fire pixel. The resolution of the data is 1km and we used the data from 2000–2012. As cloud and smoke cover reduces the confidence in satellite retrieved fire measurements, we used 95% confidence filter and used high confidence fire detections. We downloaded the country administrative datasets from Global Administrative Areas Database (www.gadm.org). The protected area boundaries were downloaded from World Database of Protected Areas [55]. The WDPA dataset also includes the IUCN categories to define the conservation status and management objectives of each protected area [56]. We used the tree canopy cover percent data from the Landsat-based 30m Global Forest Change 2000–2012 data set [49] available at http://earthenginepartners.appspot.com/science-2013-global-forest. Tree canopy cover percent is defined as canopy closure for all vegetation taller than 5m in height and is expressed as a percentage per output grid cell in the range 0–100. We used the tree cover product to demarcate the extent of forests and calculate the distance to forest edge using Euclidean distance. To determine the land cover type for Myanmar, we used MODIS Land Cover Type Yearly L3 Global 500 m SIN Grid data, also known as MCD12Q1 with the IGBP land cover classification. The data was downloaded for the year 2000 from online Data Pool at the NASA Land Processes Distributed Active Archive Center (LP DAAC), USGS/Earth Resources Observation and Science (EROS) Center (https://lpdaac.usgs.gov/data_access). Elevation was estimated from 30m ASTER data obtained from the Global Land Cover Facility (www.landcover.org). The slope and aspect was derived from the elevation data. The mean temperature for the fire season months (February to April) at 30 arc-seconds (~1 km) resolution was obtained from WorldClim [57]. We used an average of the mean temperature for the three months. Population density estimates were obtained from Socioeconomic Data and Applications Center (SEDAC). We used the Gridded Population of the World (GPW’s) v3 dataset for year 2000 at a resolution of 2.5 arc-minutes (~5 km) [58]. The road dataset was downloaded from Vector Map 0 (VMap0). The VMap0 database provides consistent, continuous global coverage of essential base map features at the largest scale. Travel time estimates were obtained from the “travel time to major cities map” developed by Nelson, 2008 [59].

## Methods

To analyze the relative contribution of each of the above causative factors and to quantify the fire susceptibility at a pixel level, we gridded the data into 1km resolution. All data were projected to the MODIS sinusoidal equal area projection. Data sets which were not 1km resolution were resampled to 1km using appropriate filters. The land cover classification data was resampled to 1 km grid using majority filter while for the percent tree cover and elevation data we used the cubic convolution filter. Specific to the population datasets, we used proportional allocation, i.e., the 5-km population data has been allocated based on proportions fitting the 1 km scale. Thus for every cell in the study area grid, we retrieved the following information: a). Tree cover percent; b). MODIS Land cover IGBP classification; c). Elevation; d). Slope; e). Aspect; f). Temperature; g). Population estimate; h). Travel time from nearest city; i). Distance to roads; j). Distance to forest edge; k). Protected/non-protected status; and l). Fire presence/absence.

We used the frequency ratio method at a pixel scale to assess the probabilistic value of each causative factor in impacting fires. This method is widely used in risk analysis studies [60], [61]. The sum of frequency ratios (FR’s) is an estimate of the probability of an event occurrence in the total study area for a given range of attribute values. For each grid cell, it is calculated as the ratio of the area where fires occurred in a specific grid cell and also, is the ratio of the probabilities of a fire occurrence to a non-occurrence for a given causative factor. The range of values extracted for each factor was divided into class intervals and the frequency ratio was calculated. The frequency ratio estimate is a quantitative indicator of the strength of the relationship between the risk event occurrence and the specific class values of the causative factor. If the estimate is higher than 1, then the probability of risk event occurrence due to the corresponding class interval values of the causative factor is considered high. If it is lower than 1, then the probability of risk event occurrence considered low. S1 and S2 Tables show the frequency ratio for each class intervals of the causative factors for both, protected and non-protected areas respectively. For each of the factors affecting the grid cell, the corresponding frequency ratios were calculated. The frequency ratios (FR’s) for all factors affecting a grid cell were then summed to arrive at fire susceptibility index. The fire susceptibility index (FSI) was calculated as:

 FSI = ∑FR (1)

Thus, each cell in our study area grid at 1km scale is associated with a fire susceptibility value which indicates the degree of fire risk in our study area. A higher FSI indicates higher fire risk. Next, we identified the highly susceptible fire pixels by protection status and ranked the factors associated with the pixels in descending order based on their FRs. Factors with higher FRs were considered dominant causative factors and vice versa.

To quantify the relative differences in protected versus non-protected areas with respect to fire incidences, we used propensity score matching technique [62]. In statistics, matching methods are often used in observational data analysis to measure the average treatment effect on the treated (ATT) group. The essence of the propensity matching method is that it identifies a control group which has similar covariates like the treated group except for the treatment (protected or non-protected). Though matching methods try to closely replicate randomization by pairing treatment and control units having similar covariates, the observational data is quasi-experimental or non-randomized by definition [63]. This gives rise to the issue of bias. Propensity score matching is a type of matching method which reduces bias due to confounding variables by using treatment and control units having covariates as similar as possible. This method is especially useful when the dimensionality of the covariates is high as it reduces the large number of matching dimensions to a single propensity score which makes the matching simpler [64]. A number of studies from various fields have used this method to evaluate the impact of treatment on target groups [34], [35], [37], [38].

One of our objectives of the study is to determine if protection by legal status makes a difference to fire occurrence. Thus the treatment in our case has been considered as the protection status and the outcome variable as the presence of fire. The treatment variable (T) is binary and T = 1 when forest is protected and T = 0 when the forest is not protected. Similarly the outcome variable is Y is fire occurrence. Y_1_ is the fire occurrence in protected areas and Y_0_ is the fire occurrence in unprotected areas. The Average Treatment Effect is difference in the outcome variable (fire occurrence) that can attributed to treatment (protection). Mathematically, the Average Treatment Effect (ATE) represented by Greek letter tau (τ) is calculated by taking an expectation over the population of interest as defined by Eq 2:

$$\;\tau \equiv E\left[Y_{1}-Y_{0}\right]$$ (2)

where *τ* = ATE, Y_1_ = outcome when exposed to treatment, Y_0_ = outcome in absence of treatment. To estimate the Average effect of Treatment on the Treated (ATT), Eq 3 is modified to reflect the estimate of Average Treatment Effect only for the treated group as shown in Eq 3.

$$\tau \equiv E\left[Y_{1}-Y_{0}|\;T\;=\;1\right]$$ (3)

For each unit in the population of interest there will be only one outcome as shown in Eq 4.

Y = Y0 when T = 0Y1 when T = 1 (4)

The propensity score is defined as a conditional probability of receiving treatment given pre-treatment characteristics (covariates) and is calculated using Eq 5 [62]:

$$p\;\left(X\right)\;=\;\mathrm{Pr}\;\left\{T\;=\;1\;|\;X\right\}$$ (5)

where T = 1 indicates exposure to treatment and T = 0 indicates not exposed to treatment. X is the vector of pre-treatment covariates. p(X) is the propensity score. The ATT can be derived from the Eqs 2 and 3 if following two conditions are satisfied.

Condition I:*T* ⏊ *X* | p(*X*): Balancing pre-treatment covariates given propensity score

Condition II:*Y*_1_,*Y*_*0*_ ⫫ *T* | p(*X*): Unconfoundedness given propensity score

Propensity score matching was particularly suitable for our study because it measures ‘conservation’ as an observable characteristic with as few parametric assumptions as possible about the model on protection effectiveness and fire occurrences. The matching package was run in R [65]. We used the Mahalanobis distance method to identify the similar matches. Matching was done with replacement and a caliper of 1 standard deviation [65].

## Results

**Fig 2:**
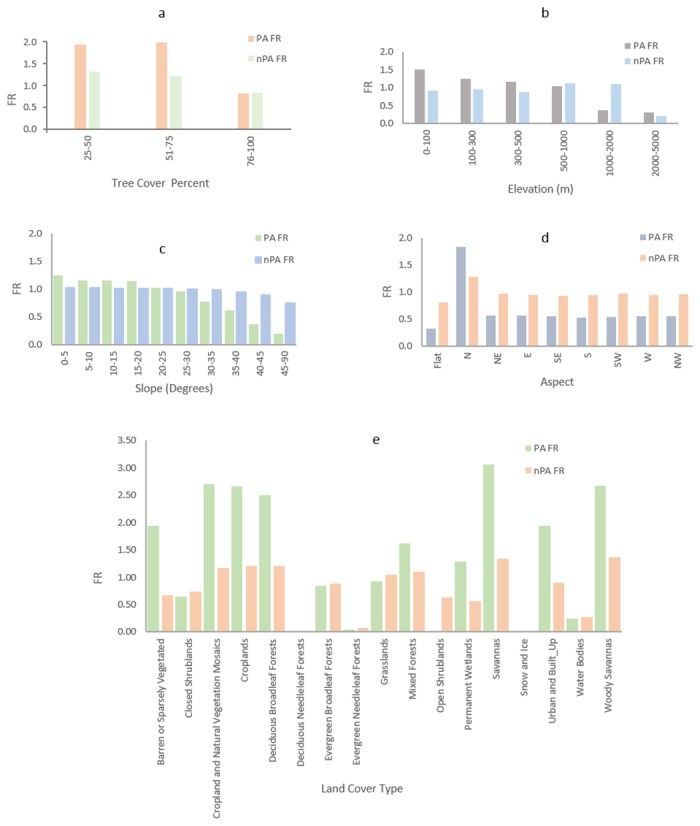
Histograms of Frequency Ratios for each causative factors in protected and non-protected forests.

The range of percent tree cover values in the forests of the study region varied from 25–100. 84% of protected areas are covered by the 75–100% tree cover category in contrast to 60% in unprotected forests. Only 13% of protected areas are covered by the 51–75% tree cover category as compared to 30% in unprotected forests. The lowest percent tree cover category (25–50%) covered an area of only 3% in protected forests compared to 10% in unprotected forests. As shown in Fig 2a, protected areas in the tree cover category ranging from 51–75% are most susceptible to fire as indicated by their high frequency ratios as compared to the protected areas in the lowest tree cover category (25–50%). However this trend is not seen in the non-protected areas, where we observed a decrease in frequency ratio with decrease in tree cover percent. In both cases, i.e., protected and non-protected areas, the highest tree cover percent category has the lowest frequency ratio indicating that forests with highest tree cover percent has least fire occurrences.

Analysis from the MODIS land cover data suggested significant variability in forest type as one moves from north/south towards center. In the north and southern regions, Myanmar is characterized by broadleaf evergreen forests, whereas the central region contain deciduous forests, shrublands and mixed forests. Protected areas are mainly dominated by broadleaf evergreen forests (79%), but include mixed forests (6%) and deciduous broadleaf forests (4%). Protected areas in the extreme north of Myanmar are mainly dominated by evergreen needleleaf forests. Outside protected areas, forests are composed of evergreen broadleaf forests (59%), woody savannas (15%), cropland and vegetation-mosaics (4%). Fig 2e reveals that protected areas on average are more prone to fire occurrences than non-protected areas as shown by their high frequency ratio values. Within protected areas, most fire occurrences were found in savannas, followed by woody savannas, cropland-natural vegetation mosaics, cropland and deciduous broadleaf forests. In non-protected areas, most fires were found in woody savannas, savannas, croplands, deciduous broadleaf forests, and cropland-vegetation mosaics.

In general, protected forests in the north and central region have higher elevation than their southern counterparts. With respect to elevation, the majority of the protected forests lie in the elevation range of 100–1000m while most of the non-protected forests lie in the elevation range of 500–2000m. As shown in Fig 2b, the probability of occurrence of fire decreases with increase in elevation in protected areas. However the same does not hold true for non-protected areas. Elevation in the range of 500–2000m show highest probability of fire occurrences in non-protected areas while lower elevation ranges i.e., 0–500m show moderate probability of fire occurrences in non-protected areas. Beyond 2000m elevation, both areas show least fire probability of fire occurrence.

The majority of the protected and non-protected forests lie in the slope range of 5–25 degrees as shown in Fig 2c. Protected areas within slope range of 0–20 degrees show high probability of fire occurrence while declines sharply as the slope increases beyond 20 degrees. The trend though similar, is not as clear for non-protected areas. Non-protected areas have a high probability of fire occurrence between 0–35 degrees and a gradual drop in fire occurrence probability was observed beyond 35 degrees slope.

Most protected areas have a northern aspect compared to unprotected forests, which dominantly had southwestern or north-eastern aspect. As shown in Fig 2d, the highest probability of fire occurrence is seen in northern aspect for both protected and non-protected areas.

The majority of protected (71%) and unprotected (81%) forests had a temperature range of 20–40°C. As expected, both protected and non-protected areas showed an increase in probability of fire occurrences with increase in temperature as seen in Fig 2f. However, compared to non-protected areas, within the temperature range of 20–30°C protected areas were more impacted by fires. This is in contrast to the lower temperature category range (0–20°C) where non-protected areas showed higher probability of fire occurrence.

In general, protected areas in north and south Myanmar have very low population density (0–25 person/ km^2^) compared to protected forests in central Myanmar where higher population densities of 75–150 persons/ km^2^ were observed. Within the range of population density of 0–300 persons/ km^2^, protected areas showed higher probability of fire occurrences than their non-protected counterparts as shown in Fig 2g.

The majority (31%) of the protected forests are at a distance of 12–24 hours from the nearest city while the majority (40%) of the unprotected forests are located at a distance of 5–10 hours from the nearest city. Further, we observed protected areas in central Myanmar being much closer to the cities than in north or south. As seen in Fig 2h, the probability of fire occurrence decreases with increase in time required to travel to the nearest cities. However, the impact of travel time on fire occurrence is more prominent in protected areas than in non-protected areas.

Our analysis revealed that only 36% of the protected forests are at a distance of 0–1km from roads compared to 61% of unprotected forests. The majority (40%) of the protected forests are at a distance of 5–50km from the road while only 14% of the unprotected forests are at the same distance from the road. Fig 2i, shows that with increase in distance from roads, the probability of fire occurrence decreases for both protected and non-protected areas. Though the frequency values of both groups are close for each category, the frequency ratio of the protected areas are consistently higher than the non-protected areas.

**Fig 3:**
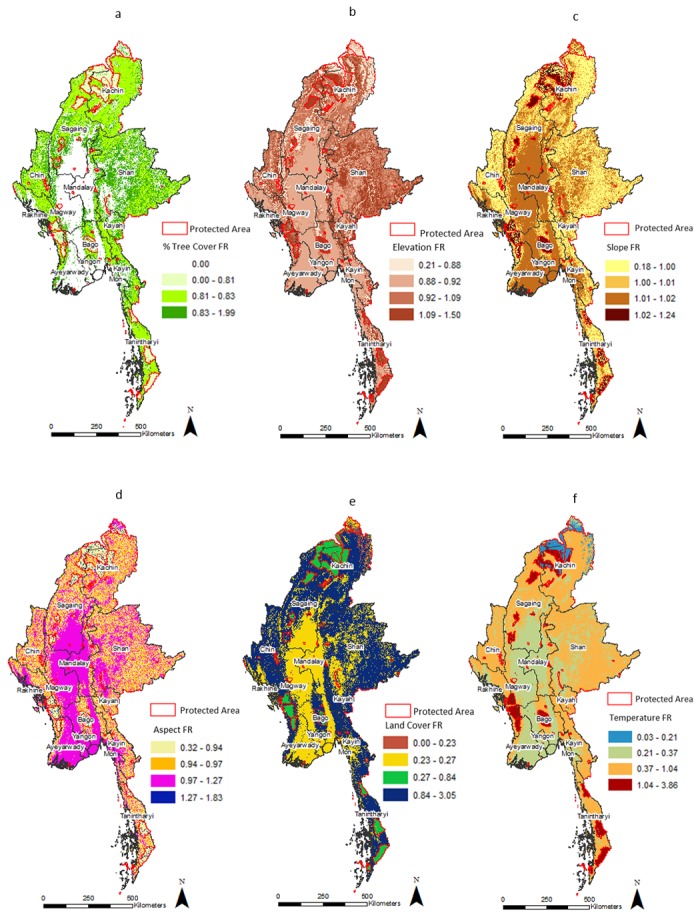
Spatial distribution of the frequency ratios (FR’s) for causative factors of fire in Myanmar.

Fig 3a shows the spatial distribution of the frequency ratios of tree cover percent. High frequency ratio values for percent tree cover were found in non-protected forests of Shan, Kayah, southern Kayin, Mon, Chin, southwestern Sagaing,and western border of Magway state. A small patch is seen in north Bago, and along the eastern borders of Magway and south Mandalay. In protected forests, high frequency ratio values for percent tree cover were found in Chatthin Wildlife Sanctuary, Alaungdaw Kathapa National Park, Maharmyaing Wildlife Sanctuary and Panlaung-Pyadalin Cave Wildlife Sanctuary.

Among the non-protected forests, high frequency ratio values for elevation follows the terrain of Myanmar as shown in Fig 3b. High frequency ratio values for elevation is found in higher elevation areas like the mountains in northern Myanmar, Chin hills, Shan plateau, Arakan Yoma, and eastern Tanintharyi while in the protected forests, it is seen in Hukuang Valley, Bumhpabum Wildlife Sanctuary, Pidaung Wildlife Sanctuary, Indawgyi Lake Wildlife Sanctuary, Chatthin Wildlife Sanctuary, Maharmyaing Wildlife Sanctuary, Panlaung-Pyadalin Cave Wildlife Sanctuary, Tanlwe-ma-e-chaung, Thandwe-chaung, Rakhine Yoma elephant range, Pegu and Bago Yoma National Parks, Kahilu Wildlife Sanctuary, Tanintharyi National Park, Lenya National Park, Pakchan Nature Reserve, and portions of Tanintharyi Nature Reserve.

Protected areas had distinctly high frequency ratio values of slope compared to the non-protected areas (Fig 3c). The highest frequency ratio of aspect was mostly found in protected areas (Fig 3d). As shown in Fig 3e, the frequency ratio of land cover was predominantly higher for non-protected forests. A few protected areas showing high frequency ratio values for land cover are Chatthin Wildlife Sanctuary, Alaungdaw Kathapa National Park, and Panlaung-Pyadalin Cave Wildlife Sanctuary. In general, protected areas had higher frequency ratio of temperature compared to non-protected areas as shown in Fig 3f. A notable exception to this trend include Hponkanrazi Wildlife Sanctuary, Khakhaborazi National Park in northern Myanmar, Natma Taung National Park in central Myanmar and portions of Hukuang Valley, portions of Bumhpabum Wildlife Sanctuary and portions of Shwe-U-Daung Wildlife Sanctuary.

The frequency ratio of distance to roads was on average less for protected areas than their non-protected counterparts. Most protected areas are located in remote regions with limited road access. Protected areas in the north and south of Myanmar and those located on the hills of central Myanmar had mostly low frequency ratio value for roads (Fig 3g). Large portions of Hukuang Valley had high frequency ratio value for roads reflecting incidents when fire was used to clear forests to build roads for infrastructure development. Similar trend reflecting anthropogenic interference is seen in the protected areas of Htamanthi Wildlife Sanctuary, Indawgyi Lake Wildlife Sanctuary, Chatthin Wildlife Sanctuary, Alaungdaw Kathapa National Park, Bago and Pegu Yoma, Kahilu Wildlife Sanctuary, foothills of Tanintharyi National Park, edges of Lenya National Park and Maharmyaing Wildlife Sanctuary. The frequency ratio of population density is shown in Fig 3h. High frequency ratio for non-protected forests occur in central Myanmar and in the state of Rakhine. Similar trend is observed in the protected forests of central Myanmar. The frequency ratio for travel time from nearest cities was an important causative factor of fire occurrrence for some protected areas like Hukuang Valley, Bago and Pegu Yoma National Parks, foothills of Tnaintharyi National Park and Tanintharyi Nature Reserve, Panlaung-Pyadalin Cave Wildlife Sanctuary, Natma Taung National Park, Chatthin Wildlife Sanctuary, Alaungdaw Kathapa National Park, Maharmyaing Wildlife Sanctuary, Shwe-U-Daung Wildlife Sanctuary. Non-protected forests along roads in south Kachin, around Huluang Valley, in state of Shan and in Tanintharyi coast were most impacted (Fig 3i).

### A) Protected areas and causative factors of fire

In protected areas of Myanmar, four important causative factors of fires were identified from the frequency analysis. They include population density, land cover type, tree cover percent and travelled time from the nearest city. Highest fire frequencies were observed for population density range of 50–75 persons/ km^2^ in protected areas. Protected areas of Chatthin Wildlife Sanctuary, Alaungdaw Kathapa National Park, parts of Maharmyaing Wildlife Sanctuary, Tanlwe-ma-e-chaung, Thandwe-chaung, Rakhine Yoma elephant range, Pegu and Bago Yoma National Parks and Tanintharyi Nature Reserve show high frequency ratio for population density. Specific to the land cover type in the protected areas, fire frequency was highest in savannas followed by woody savannas, croplands and deciduous forests. Chatthin Wildlife Sanctuary, Alaungdaw Kathapa National Park, and Panlaung-Pyadalin Cave Wildlife Sanctuary being composed of mainly of the fire susceptible land cover types showed high frequency of fire occurrences. Also, the fire frequency was highest in tree cover percent range of 51–75. Chatthin Wildlife Sanctuary and Alaungdaw Kathapa National Park showed highest frequency ratio for tree cover percent. Fig 2i shows that in protected areas, most fires occur near roads. This is in agreement with the map in Fig 3g which shows the protected areas of Hukuang Valley, Chatthin Wildlife Sanctuary and Alaungdaw Kathapa National Park most impacted by fires. Apart from the aforementioned factors, fires showed a decreasing trend with increase in elevation i.e. the highest fire frequencies were found in low elevation (0–100m) areas. In addition, highest fire frequency was found in the lower slope range (0–5 degrees). North facing slopes showed the highest fire frequencies. Further, fire frequencies increased with increasing temperature. The fire frequencies decreased with an increase in distance to roads (Fig 3i). The relative contribution of other factors is shown in (Fig 3a–3j).

### B) Non-protected areas and causative factors

Outside the protected areas, the causative factors included population density, tree cover percent and travel time from the nearest city. Non protected forests supported a higher range of population density (100–150 person/km^2^) compared to protected areas. Most of these areas are located in the valleys of central Myanmar and northern coast of Tanintharyi. We found a higher probability of fires in the tree cover range of 25–50%. Most of the high frequency ratio pixels were located in the states of Shan and Chin Hills. Compared to protected areas, the high frequency ratio of the fires in non-protected forests is concentrated in lower percent tree cover ranges of 25–50% tree cover. This indicates the impact of edge effects in the non-protected forests. Most non-protected forests were located within 2–3hrs from nearest cities, implying that accessibility to market impacts the non-protected forests. As shown in Fig 3g, forests in south Kachin and Shan are more accessible by roads show high probabilities of fire occurrence. The highest number of fires were found in 500–2000m elevation. Similar to the protected areas, the highest fire frequency was found for slope ranges from 0–5 degrees however, fire frequency was relatively high in higher slope categories (30–90 degrees slope range) than in protected areas. Similar to the protected areas, non-protected areas showed highest fire frequencies in northern slopes and increase in fire frequency with increasing temperature. The highest fire frequencies were observed for population density range of 100–150 persons/ km^2^, which is much higher than the protected areas. The fire contribution potential for each factor is shown in Fig (3a–3j).

### C) Spatial patterns of cumulative fire frequency ratio

**Fig 4:**
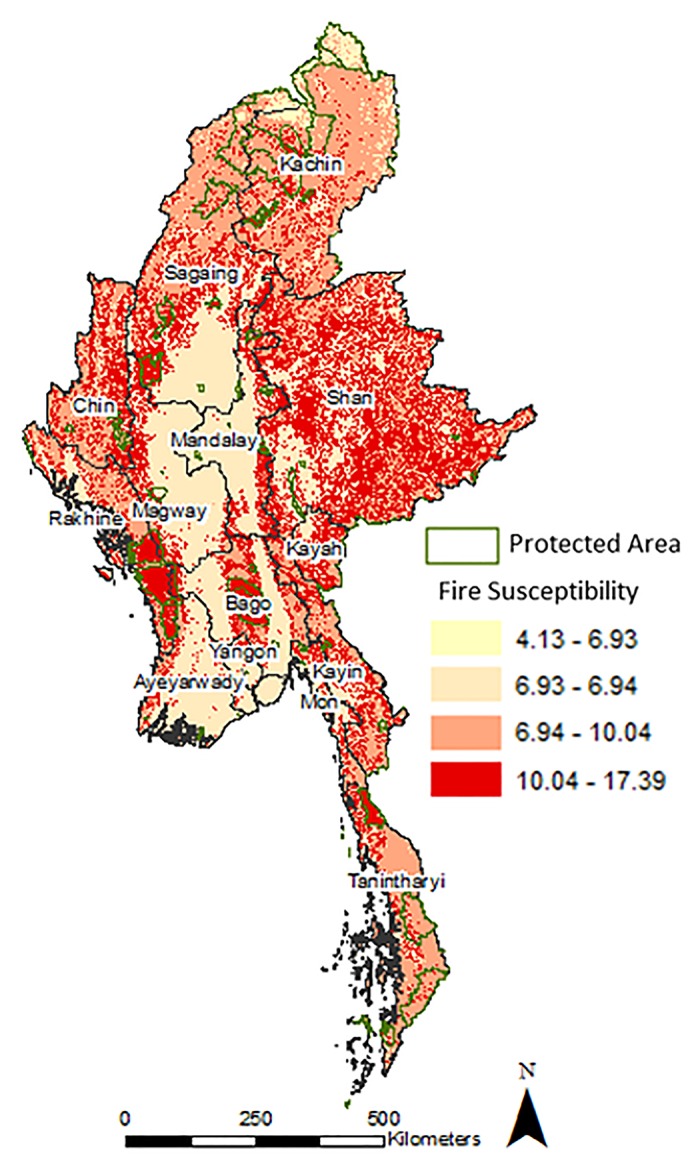
Fire Susceptibility Map of Myanmar. For a given pixel, the Fire Susceptibility was calculated by summing the individual frequency ratios for each causative factor.

The final fire susceptibility map is shown in Fig 4. The fire susceptibility analysis at the national level showed distinct spatial patterns. We found Central Myanmar to be more susceptible to fires than the northern or southern regions due to unique land cover characteristics. Central Myanmar is mostly dominated by shrublands followed by deciduous broadleaf forests and cropland-natural vegetation mosaics. At the state level, highest fire susceptibility is observed for Shan, Kayah, Kayin, Mon, central Bago, southern Rakhine, southern Kachin, southwestern Sagaing, the borders of Chin, Magway and Sagaing, northern and central Tanintharyi. Distinct clusters of fires with highest frequencies were observed in the following districts; a). Thandwein in Rakhine; b). Pegu, Taungoo and Thayarwady in Bago; c). Lashio, Kyaukme, Loiken and d). Taunggye in Shan. Fire pattern is more dispersed in the states of Kayah, Kayin, Mon, and Shan. In Tanintharyi, high fire susceptible pixels occurred mainly along the coast.

The following protected areas were highly susceptible to fire: Tanlwe-ma-e-chaung, Thandwe-chaung, Rakhine Yoma elephant range, Tanintharyi Nature Reserve, Alaungdaw Kathapa National Park, Maharmyaing Wildlife Sanctuary, Chatthin Wildlife Sanctuary, Indawgyi Wildlife Sanctuary and Pidaung Wildlife Sanctuary.

Further, in Shan most of the fires in central Loiken district occurred in the mixed forest patches of evergreen and deciduous broadleaf forests. In Kayin and Mon districts, most of the fires occurred in shrublands, woody savanna and cropland-natural vegetation mosaic classes. The fires in central Bago regions occurred primarily over Bago and Pegu Yoma districts that are mainly composed of deciduous broadleaf forests and mixed forests interspersed with evergreen broadleaf patches. Fires in the district of Thandwein, Tanlwe-ma-e-chaung, Thandwe-chaung and Rakhine Yoma elephant range occurred in evergreen broadleaf forests. Fires in north and south Myanmar were less prevalent. In Kachin, Sagaing and Tanintharyi fires mostly occurred in cropland-vegetation mosaics and evergreen broadleaf forest classes.

### D) Comparison of protected versus non-protected areas for fire occurrences

**Fig 5:**
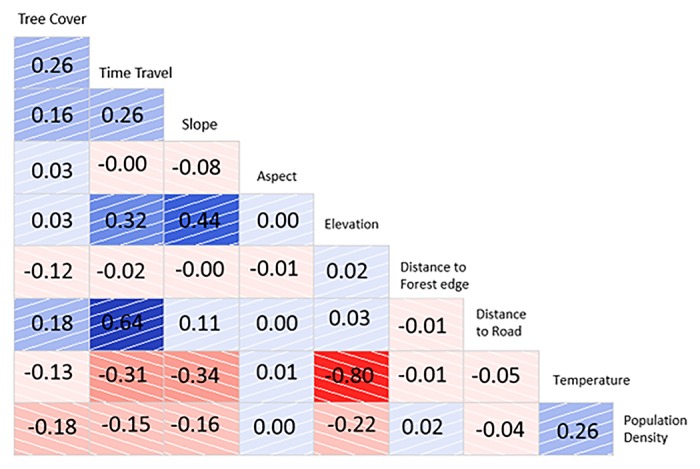
Correlogram of covariates used in propensity matching analysis. Blue indicates positive correlation and red negative correlation. Darker shades indicate stronger correlation.

A correlation analysis was done amongst different causative factors as a part of the propensity score estimation. The resultant correlograms for forest analysis are shown in Fig 5. Elevation was strongly correlated to temperature (-0.80). Since the correlations were not greater than 0.95, no collinearity was assumed. The resultant propensity score estimate is -0.1149 with standard error of 0.0024. The result is significant at 99% confidence level (t- val = -46.26, p val~0). The degree of balance achieved in the covariates while estimating the propensity score is shown in Table 1. The table shows the mean values of the treatment and control variable before and after matching. The variance ratio is a measure of the degree of balance between the treatment and control variable. Perfect balance is achieved when the variance ratio is equal to 1. In our study, after matching, the variables of tree cover percent (1.07), travel time (1.04), slope (1.09), aspect (0.97), and fire season temperature (1.04) were very well balanced with variance ratio values approaching 1. The remaining variables of elevation (1.29), distance to forest edge (0.87), distance to road (0.68) and population density (1.12) were also well balanced. The matching process greatly improved the balance of tree cover percent (from 0.59 to 1.07), travel time (from 3.65 to 1.04) and average fire season temperature (from 1.59 to 1.04). Moderate improvement was observed in the cases of elevation, slope and distance to forest edge. In summary, the propensity score analysis revealed that protected forests have 11% lower probability of fire occurrence compared to unprotected forests.

**Table 1:** Mean treatment, mean control and the variance ratio before and after matching for fire-causative factors.

Variables	Before Matching			After Matching		
	Mean Treatment	Mean Control	Variance Ratio	Mean Treatment	Mean Control	Variance Ratio
Tree Cover Percent	85.54	75.61	0.59	85.54	85.68	1.07
Travel Time	804.66	426.58	3.65	804.66	803.42	1.04
Elevation	715.14	691.93	1.56	715.14	704.76	1.29
Slope	19.85	17.61	1.23	19.85	19.71	1.09
Aspect	163.36	165.39	0.98	163.36	162.56	0.98
Distance to Forest Edge	0.53	2.71	0.15	0.53	0.65	0.87
Distance to Road	5846.30	2531.00	1.68	5846.30	6075.60	0.68
Average Fire Season Temperature	223.39	233.80	1.59	223.39	224.02	1.04
Population Density	23.20	35.13	0.31	23.20	24.61	1.12

The variance ratio is an indicator of the degree of balance achieved in the matching process. Variance ratio closer to 1 indicates better balance and robustness of the resultant propensity estimate.

## Discussion

The results of our study suggest the anthropogenic nature of fires in Myanmar. Our results are in agreement with similar studies in the region [66], [67], [68]. Moreover, analysis of the fire probabilities for the individual causative factors provides further evidence. Earlier studies [10] reported drier areas and locations with tree cover less than 60% being more prone to fires in tropical areas. We observed a similar trend in non-protected areas. However, in protected areas, fires occurred in areas of moderate tree cover. Among the land cover types, both protected and non-protected areas showed higher probability of fires in savannas and woody savannas respectively, suggesting more natural fires. Woody savannas in continental SE Asia are thought to have resulted from degradation of open forests where repeated vegetation disturbance has led to dominance of woody vegetation [14], [69], [70]. The difference of trends in fire probabilities over the elevation ranges is another major sign of anthropogenic interference. In protected areas, higher fire probability is associated with lower elevations, but in non-protected areas, higher fire probabilities were associated with mid-elevation range of 500–2000m. Further, most low elevation fires are associated with clearings near settlements while high elevation fires were associated with slash and burn agriculture (called taungya in Myanmar) practiced by the indigenous populations in the mountains [71], [72] or associated with clearing of forests for rubber plantations in recent times [42], [73]. Fires from the shifting cultivation and/or rubber plantations were found in areas outside the protected areas while such fires were totally absent in the protected areas. The fire probability in protected areas suddenly dropped after the slope varied from 25–30 degrees while the drop was more gradual for non-protected areas that occurred on higher slope ranges (30–35 degrees).

We also observed a clear influence of climate, i.e., the northern slopes were more fire prone in both protected and non-protected areas than those of the south. We attribute the climate differences to the Asian monsoon. Myanmar is mostly impacted by monsoonal climate in the south. Thus, the southern region receives more moisture than northern areas. Higher fire probability was also associated with higher temperature and population densities. This is as expected because higher air temperatures are conducive for ignition and spread of fires. Studies by [10] reported highest mean monthly burned area percent for tropical regions with a mean monthly temperature of 30°C. The same study reported an increase in burned area with an increase in population density up to 30 inhabitants per km^2^ at a global level. This value is close to the population density estimates in the protected areas, but far lower than our population density estimate outside the protected areas in Myanmar. The population density was lower in protected areas compared to non-protected areas, thus fewer fires were observed in protected areas than non-protected areas.

Accessibility from the nearest cities is a proxy for distance from markets. It was expected that areas closer to cities will have more fires than areas that are far from cities [29]. We observed a similar trend in this study, i.e., decrease in fires with increasing travel time from the nearest cities. For protected areas, most fires occurred in areas within 1–2 hours from cities, whereas in non-protected areas most fires occurred within 2–3hours from cities. The higher travel time range for non-protected areas indicate the increased anthropogenic pressure on the forest lands. Distance from roads has been found to be a major cause for forest loss [26]. For example, Wyman et al., [74] reported a decrease in deforestation by 50% with an increase in distance to roads up to 2.5km and Stolle et al.,[5] reported a 2.5 times increase in fire occurrence within a distance of 1–5km from roads. In our study, we found most fires occurring within 1km of roads in both protected and non-protected areas suggesting the anthropogenic nature of these fires. Proximity to forest edges is another indicator of the nature of ‘encroachment’ into forests. In protected areas, we found areas within 1km of the forest edge being most susceptible to fire while in non-protected areas, areas within 1–2km from forest edges were fire prone. Our results are in agreement with a similar study in the Amazon [6] where the authors found that most fires occur within 2.5 km from forest edges, while in rare events it can be as far as 5.5 km from the forest edge. The above results from frequency analysis were quite effective in delineating factors controlling vegetation fires in protected and non-protected areas of Myanmar.

Our results from propensity matching analysis show the effectiveness of protected areas in controlling vegetation fires. The protected areas had 11% less fires than the non-protected areas. Our results are comparable to a similar study conducted in Costa Rica [38] where the authors found that 10% of deforestation was avoided due to protected areas. Similar studies conducted at a regional level reported a reduction in fire incidences of 2–4% in the protected areas in Asia [34]. Overall, our results highlight spatial controls of vegetation fires in protected and non-protected areas of Myanmar. To arrive at our conclusion, we used the best available datasets at the time of publication. However in spite of our best efforts, the authors recognize the limitations of data availability and any implications it could have on our results.

## Conclusion

We investigated the biophysical and anthropogenic controls of vegetation fires in Myanmar. We used a probabilistic frequency ratio analysis to map the causative factors of fires. To analyze the effectiveness of protected areas in preventing forest fires, we used propensity matching methods. Our results suggest population density and travel time as two important anthropogenic factors impacting vegetation fires. We also mapped different biophysical fire causative factors at a 1km scale for the entire Myanmar useful for fire management. Results suggested central Myanmar being more fire prone than the southern and northern regions. Results also suggested 11% less fires in protected areas than the non-protected areas. Our study results highlight important causative factors of fires useful for understanding fire ecology and management in Myanmar.

## Supporting Information

S1 Table Frequency ratios of biophysical and anthropogenic factors for different class intervals in protected areas. (DOCX)

S2 Table Frequency ratios of biophysical and anthropogenic factors for different class intervals in non-protected areas. (DOCX)
