# Supplementary material for: Factors Controlling Vegetation Fires in Protected and Non-Protected Areas of Myanmar
Source: PLoS One. 2015 Apr 24;10(4):e0124346. doi: 10.1371/journal.pone.0124346 (PMC4409334; doi:10.1371/journal.pone.0124346)
Supplement: S1 Table — (DOCX) [file pone.0124346.s001.docx]

| **Tree Cover (%)** | **Fire**  **Observations** | **Fire**  **Observation%** | **Grids** | **Grid%** | **Frequency**  **Ratio** |
| --- | --- | --- | --- | --- | --- |
| 25-50 | 873 | 5.89 | 1740 | 3.03 | 1.94 |
| 51-75 | 3824 | 25.80 | 7422 | 12.94 | 1.99 |
| 76-100 | 10126 | 68.31 | 48199 | 84.03 | 0.81 |
| **Land Cover** | |  |  |  |  |
| Barren or Sparsely Vegetated | 1 | 0.01 | 2 | 0.00 | 1.93 |
| Closed Shrublands | 8 | 0.05 | 48 | 0.08 | 0.65 |
| Cropland and Natural Vegetation Mosaics | 307 | 2.07 | 439 | 0.77 | 2.71 |
| Croplands | 31 | 0.21 | 45 | 0.08 | 2.67 |
| Deciduous Broadleaf Forests | 1566 | 10.56 | 2423 | 4.22 | 2.50 |
| Deciduous Needleleaf Forests | 0 | 0.00 | 1 | 0.00 | 0.00 |
| Evergreen Broadleaf Forests | 10012 | 67.54 | 45633 | 79.55 | 0.85 |
| Evergreen Needleleaf Forests | 33 | 0.22 | 2884 | 5.03 | 0.04 |
| Grasslands | 10 | 0.07 | 42 | 0.07 | 0.92 |
| Mixed Forests | 1337 | 9.02 | 3207 | 5.59 | 1.61 |
| Open Shrublands | 0 | 0.00 | 89 | 0.16 | 0.00 |
| Permanent Wetlands | 111 | 0.75 | 335 | 0.58 | 1.28 |
| Savannas | 15 | 0.10 | 19 | 0.03 | 3.06 |
| Snow and Ice | 0 | 0.00 | 15 | 0.03 | 0.00 |
| Urban and Built_Up | 2 | 0.01 | 4 | 0.01 | 1.93 |
| Water Bodies | 11 | 0.07 | 178 | 0.31 | 0.24 |
| Woody Savannas | 1379 | 9.30 | 1997 | 3.48 | 2.67 |
| **Elevation (m)** |  |  |  |  |  |
| 0-100 | 1490 | 10.05 | 3857 | 6.69 | 1.50 |
| 101-300 | 4842 | 32.66 | 15186 | 26.35 | 1.24 |
| 301-500 | 3559 | 24.00 | 11915 | 20.67 | 1.16 |
| 501-1000 | 3802 | 25.65 | 14096 | 24.46 | 1.0487 |
| 1001-2000 | 788 | 5.32 | 8357 | 14.50 | 0.37 |
| 2001-5000 | 342 | 2.31 | 4220 | 7.32 | 0.32 |
| **Slope (⁰)** |  |  |  |  |  |
| 0-5 | 1671 | 11.27 | 5194 | 9.05 | 1.25 |
| 6-10 | 2796 | 18.86 | 9341 | 16.28 | 1.16 |
| 11-15 | 2747 | 18.53 | 9208 | 16.05 | 1.15 |
| 16-20 | 2491 | 16.80 | 8472 | 14.77 | 1.14 |
| 21-25 | 1982 | 13.37 | 7487 | 13.05 | 1.02 |
| 26-30 | 1491 | 10.06 | 6030 | 10.51 | 0.96 |
| 31-35 | 871 | 5.88 | 4324 | 7.54 | 0.78 |
| 36-40 | 476 | 3.21 | 2975 | 5.19 | 0.62 |
| 41-45 | 179 | 1.21 | 1900 | 3.31 | 0.36 |
| 46-90 | 119 | 0.80 | 2430 | 4.24 | 0.19 |
| **Aspect** |  |  |  |  |  |
| Flat | 2 | 0.01 | 13 | 0.02 | 0.32 |
| N | 23490 | 64.53 | 27011 | 35.17 | 1.83 |
| NE | 3001 | 8.24 | 11353 | 14.78 | 0.56 |
| E | 1452 | 3.99 | 5416 | 7.05 | 0.57 |
| SE | 1113 | 3.06 | 4259 | 5.55 | 0.55 |
| S | 1819 | 4.99 | 7321 | 9.53 | 0.52 |
| SW | 2930 | 8.05 | 11491 | 14.96 | 0.54 |
| W | 1485 | 4.08 | 5638 | 7.34 | 0.56 |
| NW | 1111 | 3.05 | 4298 | 5.59 | 0.55 |
| **Temperature (⁰C)** | |  |  |  |  |
| 0-10 | 57 | 0.38 | 2029 | 3.54 | 0.11 |
| 11-20 | 784 | 5.29 | 14429 | 25.15 | 0.21 |
| 21-30 | 13981 | 94.39 | 40902 | 71.31 | 1.32 |
| >30 | 1 | 0.01 | 1 | 0.01 | 3.87 |
| **Population Density (person/km^2^)** |  |  |  |  |  |
| 0-25 | 3802 | 25.65 | 38894 | 67.81 | 0.38 |
| 26-50 | 7070 | 47.70 | 11688 | 20.38 | 2.34 |
| 51-75 | 354 | 2.39 | 469 | 0.82 | 2.92 |
| 76-100 | 1537 | 10.37 | 2932 | 5.11 | 2.03 |
| 101-150 | 1560 | 10.52 | 2488 | 4.34 | 2.43 |
| 151-200 | 462 | 3.12 | 756 | 1.32 | 2.36 |
| 201-250 | 30 | 0.20 | 121 | 0.21 | 0.96 |
| 251-300 | 8 | 0.05 | 13 | 0.02 | 2.38 |
| 301-500 | 0 | 0 | 0 | 0 | 0.00 |
| 501-1000 | 0 | 0 | 0 | 0 | 0.00 |
| 1001-12000 | 0 | 0 | 0 | 0 | 0.00 |
| **Travel Time (mins)** |  | |  |  |  |
| 0-60 | 135 | 0.91 | 274 | 0.48 | 1.90 |
| 61-120 | 860 | 5.80 | 1529 | 2.67 | 2.18 |
| 121-180 | 1340 | 9.04 | 2569 | 4.48 | 2.02 |
| 181-240 | 1427 | 9.63 | 3115 | 5.43 | 1.77 |
| 241-300 | 1436 | 9.69 | 3392 | 5.91 | 1.63 |
| 301-600 | 4942 | 33.34 | 16824 | 29.33 | 1.13 |
| 601-720 | 969 | 6.54 | 4650 | 8.11 | 0.81 |
| 721-1440 | 3266 | 22.03 | 17639 | 30.76 | 0.71 |
| 1441-2880 | 444 | 2.99 | 6578 | 11.47 | 0.26 |
| 2881-4320 | 4 | 0.03 | 776 | 1.35 | 0.02 |
| **Distance to Roads (m)** |  | |  |  |  |
| 0 - 1000 | 6338 | 42.50 | 20852 | 36.35 | 1.17 |
| 1001 - 2000 | 1110 | 7.44 | 4030 | 7.03 | 1.06 |
| 2001 - 3000 | 1011 | 6.78 | 3721 | 6.49 | 1.05 |
| 3001 - 4000 | 728 | 4.88 | 2969 | 5.18 | 0.94 |
| 4001 - 5000 | 765 | 5.13 | 2919 | 5.09 | 1.01 |
| 5001 - 10000 | 2309 | 15.48 | 10217 | 17.81 | 0.87 |
| 10001 - 50000 | 2652 | 17.78 | 12559 | 21.89 | 0.81 |
| 50001 - 100000 | 0 | 0 | 94 | 0.16 | 0.00 |
| **Distance to forest edge (m)** | | |  |  |  |
| 0 - 1000 | 14823 | 100 | 57359 | 99.99 | 1.00 |
| 1001 - 2000 | 0 | 0 | 2 | 0.00 | 0.00 |
| 2001 - 3000 | 0 | 0 | 0 | 0 | 0.00 |
