# Supplementary material for: Factors Controlling Vegetation Fires in Protected and Non-Protected Areas of Myanmar
Source: PLoS One. 2015 Apr 24;10(4):e0124346. doi: 10.1371/journal.pone.0124346 (PMC4409334; doi:10.1371/journal.pone.0124346)
Supplement: S2 Table — (DOCX) [file pone.0124346.s002.docx]

| **Tree Cover (%)** | **Fire**  **Observations** | **Fire**  **Observation%** | **Grids** | **Grid%** | **Frequency**  **Ratio** |
| --- | --- | --- | --- | --- | --- |
| 25-50 | 27337 | 12.90 | 37083 | 9.75 | 1.32 |
| 51-75 | 79400 | 37.46 | 116433 | 30.61 | 1.22 |
| 76-100 | 105250 | 49.65 | 226902 | 59.65 | 0.83 |
| **Land Cover** | |  |  |  |  |
| Barren or Sparsely Vegetated | 15 | 0.01 | 40 | 0.01 | 0.67 |
| Closed Shrublands | 153 | 0.07 | 371 | 0.10 | 0.74 |
| Cropland and Natural Vegetation Mosaics | 9823 | 4.63 | 15218 | 4.00 | 1.15 |
| Croplands | 1256 | 0.59 | 1862 | 0.49 | 1.21 |
| Deciduous Broadleaf Forests | 23169 | 10.93 | 34419 | 9.05 | 1.21 |
| Deciduous Needleleaf Forests | 0 | 0.00 | 2 | 0.00 | 0.00 |
| Evergreen Broadleaf Forests | 111957 | 52.81 | 227859 | 59.90 | 0.88 |
| Evergreen Needleleaf Forests | 208 | 0.10 | 5375 | 1.41 | 0.07 |
| Grasslands | 326 | 0.15 | 564 | 0.15 | 1.04 |
| Mixed Forests | 19644 | 9.27 | 32233 | 8.47 | 1.09 |
| Open Shrublands | 12 | 0.01 | 34 | 0.01 | 0.63 |
| Permanent Wetlands | 725 | 0.34 | 2300 | 0.60 | 0.57 |
| Savannas | 605 | 0.29 | 808 | 0.21 | 1.34 |
| Snow and Ice | 0 | 0.00 | 1 | 0.00 | 0.00 |
| Urban and Built_Up | 72 | 0.03 | 144 | 0.04 | 0.89 |
| Water Bodies | 267 | 0.13 | 1762 | 0.46 | 0.27 |
| Woody Savannas | 43755 | 20.64 | 57426 | 15.10 | 1.36 |
| **Elevation (m)** |  |  |  |  |  |
| 0-100 | 20109 | 9.49 | 39181 | 10.29 | 0.92 |
| 101-300 | 43946 | 20.73 | 82010 | 21.56 | 0.96 |
| 301-500 | 25912 | 12.22 | 52691 | 13.85 | 0.88 |
| 501-1000 | 66370 | 31.31 | 106834 | 28.08 | 1.11 |
| 1001-2000 | 54358 | 25.64 | 88744 | 23.32 | 1.09 |
| 2001-5000 | 1292 | 0.61 | 10958 | 2.88 | 0.21 |
| **Slope (⁰)** |  |  |  |  |  |
| 0-5 | 25637 | 12.09 | 45102 | 11.86 | 1.02 |
| 6-10 | 40361 | 19.04 | 71051 | 18.68 | 1.02 |
| 11-15 | 36587 | 17.26 | 64959 | 17.08 | 1.01 |
| 16-20 | 32022 | 15.11 | 56902 | 14.96 | 1.01 |
| 21-25 | 27031 | 12.75 | 48063 | 12.63 | 1.01 |
| 26-30 | 20506 | 9.67 | 36826 | 9.68 | 0.99 |
| 31-35 | 14101 | 6.65 | 25610 | 6.73 | 0.99 |
| 36-40 | 8448 | 3.99 | 16112 | 4.24 | 0.94 |
| 41-45 | 4378 | 2.07 | 8744 | 2.29 | 0.89 |
| 46-90 | 2916 | 1.37 | 7049 | 1.85 | 0.74 |
| **Aspect** |  |  |  |  |  |
| Flat | 68 | 0.03 | 144 | 0.04 | 0.81 |
| N | 40000 | 17.90 | 53862 | 14.05 | 1.27 |
| NE | 42080 | 18.83 | 74149 | 19.34 | 0.97 |
| E | 19755 | 8.84 | 35793 | 9.34 | 0.94 |
| SE | 14632 | 6.55 | 26941 | 7.03 | 0.93 |
| S | 25216 | 11.28 | 45662 | 11.91 | 0.95 |
| SW | 42664 | 19.09 | 76007 | 19.83 | 0.96 |
| W | 21923 | 9.81 | 39789 | 10.38 | 0.95 |
| NW | 17114 | 7.66 | 30933 | 8.07 | 0.95 |
| **Temperature (⁰C)** | |  |  |  |  |
| 0-10 | 487 | 0.23 | 4747 | 1.25 | 0.18 |
| 11-20 | 32199 | 15.19 | 67519 | 17.75 | 0.86 |
| 21-30 | 179122 | 84.50 | 307962 | 80.95 | 1.04 |
| >30 | 179 | 0.08 | 190 | 0.05 | 1.69 |
| **Population Density (person/ km^2^)** |  |  |  |  |  |
| 0-25 | 115567 | 54.52 | 211686 | 55.65 | 0.98 |
| 26-50 | 52855 | 24.93 | 93145 | 24.49 | 1.02 |
| 51-75 | 14981 | 7.07 | 24576 | 6.46 | 1.09 |
| 76-100 | 9090 | 4.29 | 18599 | 4.89 | 0.88 |
| 101-150 | 13333 | 6.29 | 20777 | 5.46 | 1.15 |
| 151-200 | 3659 | 1.73 | 7151 | 1.88 | 0.91 |
| 201-250 | 1574 | 0.74 | 2769 | 0.73 | 1.02 |
| 251-300 | 306 | 0.14 | 492 | 0.13 | 1.11 |
| 301-500 | 566 | 0.27 | 1073 | 0.28 | 0.95 |
| 501-1000 | 47 | 0.02 | 134 | 0.04 | 0.63 |
| 1001-12000 | 7 | 0.00 | 14 | 0.00 | 0.90 |
| **Travel Time (mins)** | |  |  |  |  |
| 0-60 | 3955 | 1.87 | 6868 | 1.81 | 1.03 |
| 61-120 | 16547 | 7.81 | 25344 | 6.66 | 1.17 |
| 121-180 | 25345 | 11.96 | 37628 | 9.89 | 1.21 |
| 181-240 | 29452 | 13.89 | 44925 | 11.81 | 1.17 |
| 241-300 | 26748 | 12.62 | 43746 | 11.50 | 1.10 |
| 301-600 | 84192 | 39.72 | 149408 | 39.27 | 1.01 |
| 601-720 | 11283 | 5.32 | 24270 | 6.38 | 0.83 |
| 721-1440 | 13690 | 6.46 | 40340 | 10.60 | 0.61 |
| 1441-2880 | 773 | 0.36 | 7794 | 2.05 | 0.18 |
| 2881-4320 | 2 | 0.00 | 95 | 0.02 | 0.03 |
| **Distance to Roads (m)** | |  |  |  |  |
| 0 - 1000 | 139905 | 65.99 | 231111 | 60.75 | 1.09 |
| 1001 - 2000 | 19400 | 9.15 | 34395 | 9.04 | 1.01 |
| 2001 - 3000 | 15621 | 7.37 | 28649 | 7.53 | 0.98 |
| 3001 - 4000 | 9275 | 4.38 | 18224 | 4.79 | 0.91 |
| 4001 - 5000 | 7551 | 3.56 | 15414 | 4.05 | 0.88 |
| 5001 - 10000 | 15430 | 7.28 | 34140 | 8.97 | 0.81 |
| 10001 - 50000 | 4635 | 2.19 | 17782 | 4.67 | 0.47 |
| 50001 - 100000 | 170 | 0.08 | 703 | 0.18 | 0.43 |
| **Distance to forest edge (m)** | | |  |  |  |
| 0 - 1000 | 211936 | 99.98 | 380360 | 99.98 | 0.99 |
| 1001 - 2000 | 34 | 0.01 | 39 | 0.01 | 1.56 |
| 2001 - 3000 | 8 | 0.00 | 10 | 0.00 | 1.44 |
